# Micro-scaled topographies direct differentiation of human epidermal stem cells

**DOI:** 10.1016/j.actbio.2018.12.003

**Published:** 2019-01-15

**Authors:** Sebastiaan Zijl, Aliaksei S. Vasilevich, Priyalakshmi Viswanathan, Ayelen Luna Helling, Nick R.M. Beijer, Gernot Walko, Ciro Chiappini, Jan de Boer, Fiona M. Watt

**Affiliations:** a Centre for Stem Cells and Regenerative Medicine, King’s College London, 28th Floor, Tower Wing, Guy’s Hospital, Great Maze Pond, London SE1 9RT, United Kingdom; b Department of Biomedical Engineering, Eindhoven University of Technology, Eindhoven, The Netherlands; c Department of Biology and Biochemistry, University of Bath, United Kingdom; d Centre for Craniofacial and Regenerative Biology, Dental Institute, King's College London, 27th Floor, Tower Wing, Guy’s Hospital, Great Maze Pond, London SE1 9RT, United Kingdom; e Materiomics bv, Maastricht, The Netherlands

**Keywords:** Topography, Keratinocytes, Epidermal stem cells, Actin polymerization, Differentiation, Actomyosin contractility

## Abstract

Human epidermal stem cells initiate terminal differentiation when spreading is restricted on ECM-coated micropatterned islands, soft hydrogels or hydrogel-nanoparticle composites with high nanoparticle spacing. The effect of substrate topography, however, is incompletely understood. To explore this, primary human keratinocytes enriched for stem cells were seeded on a topographical library with over 2000 different topographies in the micrometre range. Twenty-four hours later the proportion of cells expressing the differentiation marker transglutaminase-1 was determined by high content imaging. As predicted, topographies that prevented spreading promoted differentiation. However, we also identified topographies that supported differentiation of highly spread cells. Topographies supporting differentiation of spread cells were more irregular than those supporting differentiation of round cells. Low topography coverage promoted differentiation of spread cells, whereas high coverage promoted differentiation of round cells. Based on these observations we fabricated a topography in 6-well plate format that supported differentiation of spread cells, enabling us to examine cell responses at higher resolution. We found that differentiated spread cells did not assemble significant numbers of hemidesmosomes, focal adhesions, adherens junctions, desmosomes or tight junctions. They did, however, organise the actin cytoskeleton in response to the topographies. Rho kinase inhibition and blebbistatin treatment blocked the differentiation of spread cells, whereas SRF inhibition did not. These observations suggest a potential role for actin polymerization and actomyosin contraction in the topography-induced differentiation of spread cells.

**Statement of Significance:**

The epidermis is the outer covering of the skin. It is formed by layers of cells called keratinocytes. The basal cell layer contains stem cells, which divide to replace cells in the outermost layers that are lost through a process known as differentiation. In this manuscript we have developed surfaces that promote the differentiation of epidermal stem cells in order to understand the signals that control differentiation. The experimental tools we have developed have the potential to help us to devise new treatments that control diseases such as psoriasis and eczema in which epidermal stem cell proliferation and differentiation are disturbed.

## Graphical abstract

**Figure:**
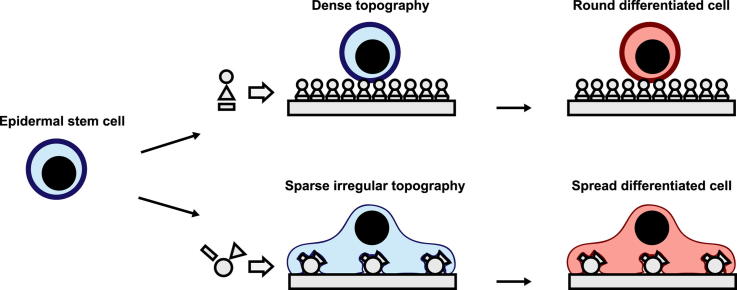

## 1 Introduction

Human skin is comprised of two layers: the dermis, which is a well vascularised connective tissue, and the epidermis, which is formed of multiple layers of epithelial cells called keratinocytes. While the dermis gives the skin its flexibility and strength, the main functions of the epidermis are to prevent water loss and to protect the body from external threats such as infections and mechanical stress [1]. Within the epidermis, the basal layer contains stem cells that self-renew and generate daughter cells that undergo terminal differentiation as they move through the suprabasal layers towards the tissue surface [2], [3].

Human epidermal cells (keratinocytes) can be grown *in vitro* under conditions that support both the maintenance of the stem cell compartment and the generation of terminally differentiated cells. Markers that enrich for stem cells include extracellular matrix (ECM) receptors of the integrin family (such as integrin-β1 and β4), whereas markers of terminal differentiation include involucrin (IVL) and transglutaminase-1 (TGM1), cytoplasmic proteins that contribute to epidermal barrier formation. Cultured human epidermis can be used to examine, at single cell resolution, how stem cells make fate decisions based on signalling cues from the external microenvironment [2], [4] and can be used to repair damaged human skin [5].

Previous studies have highlighted the role of cell-substrate interactions in controlling exit from the human epidermal stem cell compartment [6], [7]. When single cells are seeded on ECM-coated micro-patterned islands, differentiation is triggered by restricted spreading, which is dependent on the ratio of F- to G-actin and activation of serum respose factor (SRF) [6]. Differentiation is also triggered when cells are plated on ECM coated soft hydrogels or hydrogel-nanoparticle composites with high nanoparticle spacing. On the latter, cells fail to spread but differentiation is not triggered by SRF activation. Instead, differentiation is linked to downregulation of extracellular signal-regulated kinase (ERK)/mitogen-activated protein kinase (MAPK) activity caused by failed integrin clustering [7]. Thus, different extracellular cues can trigger differentiation via different intracellular signalling routes.

Little is known about the effects of micron-scale substrate topography on epidermal differentiation. To investigate the effect of topography on human epidermal stem cells, we focused on a library of micron-scale topographies, known as the TopoChip, which has been used previously to identify topographies that regulate the behaviour of other cell types [8], [9]. This platform allows for the screening of a large number of different topographical features using small numbers of cells. We used the TopoChip platform to screen for the effect of micro-topography on keratinocyte behaviour *in vitro*. We show that differentiation is not obligatorily linked to restricted spreading and that specific topographies of interest can be scaled up in a 6-well plate setting for mechanistic studies.

## 2 Methods

### 2.1 TopoChip fabrication

The TopoChip library was prepared as reported previously [8] and comprised 2175 different surface topographies and a flat control, printed on a polystyrene (PS) chip. Topographies were generated based on a random *in silico* combination of primitive shapes (circles, triangles, rectangles). Each individual TopoUnit (dimensions: 300 × 300 μm) contained a different kind of topography (composed of different primitive shapes). Different topographies not only varied in shape, but also, amongst other characteristics, in overall size, coverage and regularity. Each chip (dimensions: 2 × 2 cm^2^, 66 × 66 TopoUnits) contained internal duplicates for every TopoUnit. The location of each TopoUnit was the same on every TopoChip. To rule out location bias, duplicate arrays were placed diagonally to each other. TopoChips were made from PS by hot embossing PS films (Goodfellow) [10]. Prior to cell culture, TopoChips were treated with oxygen plasma for 1 min or air plasma for 2 min (Zepto low cost plasma cleaner, Diener electronic) and sterilised for 5 min in 70% ethanol. When not directly used, TopoChips were stored dry and used within 6 months.

### 2.2 Fabrication of polystyrene topographies in 6-well plate format

Topography surfaces chosen for validation (based on TopoUnits) were made using soft lithography [11]. To do this, a silicon (Si) wafer template was fabricated (Kelvin Nanotech), coated with polydimethylsiloxane (PDMS) and cured (>5h at 80 °C) to create a negative mould of the topographies. The latter was coated with polystyrene (PS) to recreate the initial topographies present on the wafer. To do this, the same PS films as used for the TopoChips (Goodfellow) were dissolved in the solvent γ-butyrolactone (GBL). To obtain pure PS, GBL was next evaporated on a hot plate in a fume hood (4 h at 95 °C, followed by >12 h at 150 °C), leaving only the solidified PS behind on the PDMS mould [11]. After coating, PDMS moulds were peeled off the PS topographies, which were then prepared for cell culture. This was done as described for TopoChips.

### 2.3 Cell culture

Primary human keratinocytes (NHKs, strain Km or Kp) were obtained from surgically discarded normal neonatal human foreskin with appropriate ethical consent. NHKs in all experiments were used at passage 2–8. J2-3T3 cells were originally obtained from Dr. James Rheinwald (Department of Dermatology, Harvard Skin Research Centre, USA) and were used at passage 3–12. All cells were regularly tested for mycoplasma and were negative. For routine culture, NHKs were cultured in FAD medium (Gibco), comprising 1 part Ham’s F12 medium and 3 parts Dulbecco's Modified Eagle Medium (DMEM) supplemented with 10^−4^ M adenine, and 10% (v/v) foetal bovine serum (FBS), 0.5 μg/ml hydrocortisone, 5 μg/ml insulin, 10^−10^ M cholera toxin, 10 ng/ml epidermal growth factor (EGF), 100 IU/ml penicillin and 100 μg/ml streptomycin (complete FAD medium). NHKs were cultured on mitotically inactivated (4 μg/ml mitomycin C treatment for 2.5–3 h, Sigma) J2-3T3 cells (feeder cells) as previously described [12], [13]. Feeder cells were cultured in high-glucose containing DMEM medium (Sigma or Gibco), supplemented with 100 IU/ml penicillin, 100 μg/ml streptomycin and 10% (v/v) adult bovine serum (Life Technologies).

For experiments, NHKs were harvested at 70–80% confluence and collected by trypsinization (0.05% trypsin in EDTA, Gibco) after removal of the feeder cells by Versene (Gibco) treatment. After trypsinization, NHKs were filtered twice through a 40 μm cell strainer (Falcon) and re-seeded onto TopoChips or home-made PS topographies (5 × 10^4^ cells/cm^2^) in complete FAD medium. To enrich for stem cells, cells were allowed to attach for 45 min to 1 h at 37 °C. Non-adherent cells were rinsed off by washing 3–5 times with fresh medium [12]. Cells were fixed after stem cell adhesion or 24 h later.

### 2.4 Pharmacological inhibitors

For inhibitor studies, cells were allowed to adhere to the substrates as usual (45 min to 1 h), after which drugs were added to the medium for an additional 24 h. Rho-kinase inhibitor Y-27632 (ROCKi, Enzo) was used (10 μM) to inhibit Rho-associated protein kinase (ROCK). Blebbistatin (Sigma, 25 μM) was used to inhibit Myosin II, and thus actomyosin contractility. CCG-1423 (Merck, 1 μM) was used to inhibit the activity of serum response factor (SRF). All inhibitors were dissolved in Dimethyl sulfoxide (DMSO), such that the final concentration of DMSO in the medium was 0.1%. All drugs were compared to a vehicle control with 0.1% DMSO only. After 24 h, cells were analysed for the expression of differentiation markers (TGM1 and/or IVL) by immunofluorescence.

### 2.5 Immunofluorescence microscopy

Cells grown on TopoChips or PS substrates were typically fixed in 4% (w/v) paraformaldehyde (PFA, Sigma) for 15 min at room temperature. For desmosome staining, cells were fixed in a mixture of 50% methanol and 50% acetone (Sigma or Acros organics) for 10 min at −20 °C. PFA fixed samples were permeabilized with 0.2% (v/v) Triton X-100 (Tx100, Sigma) for 10 min at room temperature. For integrin-β4 staining, cells were fixed and permeabilized simultaneously (in 4% PFA containing 0.2% Tx100 for 15 min). Fixed cells were blocked for 1 h at room temperature in PBS containing 10% (v/v) FBS (Sigma) and 0.25% (v/v) cold water fish skin gelatin (Sigma). Primary antibodies (Table S1) were diluted in blocking buffer and incubated for 1 h at room temperature or overnight at 4 °C [14].

Samples stained with primary antibodies were washed with PBS and incubated with Alexa Fluor-conjugated secondary antibodies (Alexa Fluor 488, 594 or 647) for 1 h at room temperature. Phalloidin (typically Alexa Fluor 488 or 594; to label F-actin) and 4′,6-diamidino-2-phenylindole (DAPI, Thermo Fisher Scientific; to label nuclei) were included in the secondary antibody mix where indicated to label filamentous actin and nuclei, respectively. Prior to imaging, TopoChips were mounted onto glass 6-well plates (Cellvis) with ProLong Gold anti-fade reagent (Thermo Fisher Scientific). Images (20× objective) were acquired on an Operetta high-content imaging system (Perkin Elmer) or an inverted Eclipse Ti-E microscope (Nikon). For high resolution imaging (63× objective), images were acquired on an inverted Eclipse Ti-E confocal microscope (Nikon). To reduce imaging times on the Operetta, TopoChips were typically imaged in quarters. Automated image analysis was performed using Harmony software (Perkin Elmer) or Fiji/ImageJ. Cells were identified based on DAPI and F-actin staining, before the analysis of differentiation markers (TGM1 and IVL). Complete image analysis pipelines can be provided upon request.

### 2.6 Real time quantitative RT-PCR analysis

RNA was isolated from keratinocytes using the RNeasy kit (Qiagen). Complementary DNA was generated with the QuantiTect Reverse Transcription kit (Qiagen). Quantitive (q)RT-PCR analysis was performed using qRT-PCR primers and Fast SYBR green master mix (Life Technologies). QRT-PCR reactions were run on a CFX384 Real-Time System (Bio-Rad) with RPL-13, GAPDH and TBP as housekeeping genes for normalization. Relative fold expression levels of relevant genes were calculated using the Livak method [15]. The following qPCR oligos sequences (forward and reverse sequences) were used: GCCTCAGCCTTACTGTGAGT and TGTTTCATTTGCTCCTGATGG (Involucrin), AAGGCCGGATGCCAGTTTAG and TGCTGAATGGCAACCATCAAA (Suprabasin), TGGAGGACTACCGCCGGTTCC and CGGTGACGGCCAGCCGTTTT (Envoplakin), GCAGAGTGACCTGGCTCGGCT and GCCGCATCCGCCTCTAGCAC (Periplakin), AACAGCTCATGAGGCTACG and AACAATGGAGGAAGGGCAGG (RPL-13), GAAGAGAGAGACCCTCACTGCTG and ACTGTGAGGAGGGGAGATTCAGT (GAPDH), GTGACCCAGCATCACTGTTTC and GAGCATCTCCAGCACACTCT (TBP).

### 2.7 Reproducibility and statistics

For every experimental replicate 800–1100 individual TopoUnits were analysed per quarter TopoChip. Results are from 5 independent experiments on different TopoChips, unless stated otherwise. Experiments on home-made PS substrates were performed at least three times in independent experiments. To classify TopoChip topographies that had a positive or negative effect on spreading and differentiation we used binary classification tree algorithms from the “rpart” package, implemented in R (version 3.1.223). Before training the predictive models, all highly correlated features with r^2^ greater than 0.75 were eliminated from further analysis. To create the models, we used 75% of the TopoUnits and tested the accuracy of the model on the remaining 25% of the TopoUnits. Models were trained with 10-fold cross validation in the “caret” package and the classification trees were visualized using the “party” package. These techniques for classifying topographies have been published previously [8], [9], [16].

## 3 Results

### 3.1 Topography affects the morphology of differentiating keratinocytes

**Fig. 1:**
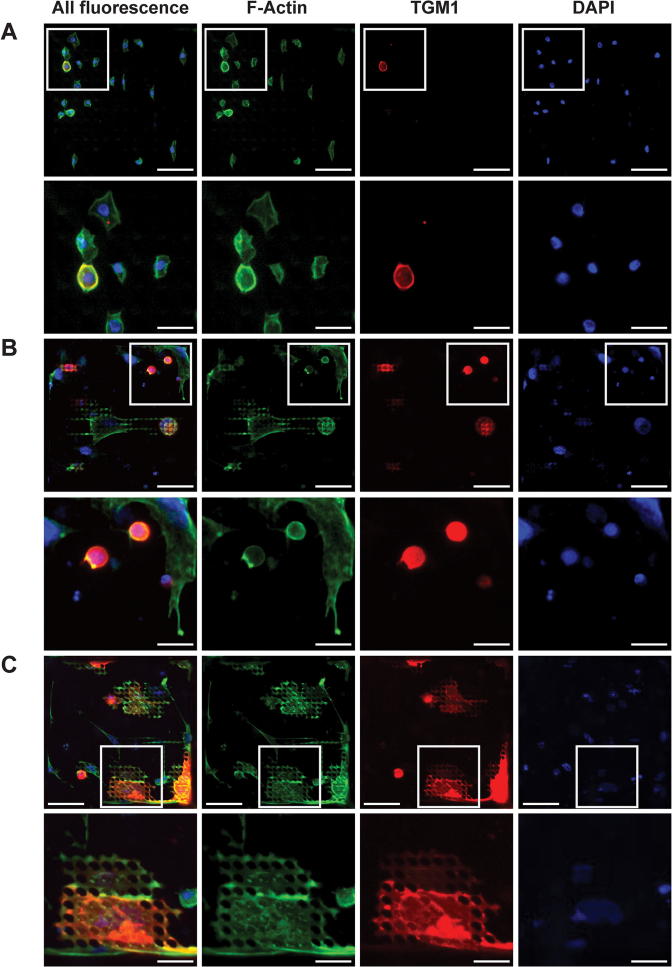
Differentiation of round and spread keratinocytes on TopoChips. A) Example of a TopoUnit after stem cell adhesion (1 h). Panels represent staining for F-Actin (green), TGM1 (red) and DAPI (blue). B-C) Examples of TopoUnits where keratinocytes were cultured for an additional 24 h. Here, cells differentiated as either B) round or C) spread cells. The first column represents composite images of all fluorescent channels. The last three columns depict individual channel images for F-actin (green), TGM1 (red) and DAPI (blue). Boxed regions in individual panels are shown at higher magnification below. Results are representative images from five independent experiments. Scale bars represent 50 μm (upper rows in A-C) or 20 μm (lower rows). Images were obtained using an automated high content imaging microscope (Operetta), with a 20× objective. TGM1: transglutaminase-1.

TopoChips are microchips containing a library of 2175 different surface topographies and a flat control substrate. Individual topographies were printed in a 300 × 300 μm well (TopoUnit) and duplicated on a different location of the chip. Topographies were made based on a random *in silico* combination of primitive topographical shapes (circles, triangles and rectangles) [8]. This provides topographies of various shapes, densities and regularities and thus represents an unbiased tool to study the effect of surface topography on cell behaviour [8]. TopoChips were fabricated by hot embossing PS films that are similar to tissue culture plastic [10]. To evaluate the response of keratinocytes to different topographies, primary human keratinocytes (strain Km or Kp) from normal neonatal human foreskin (NHKs) were plated on the TopoChips at a density of 5 × 10^4^ cells per cm^2^. To enrich for stem cells, NHKs were allowed to adhere for 45 min to 1 h at 37 °C. Non-adherent differentiated cells (expressing lower levels of integrins) were removed by washing [12]. After adhesion, typically five to twenty cells attached per TopoUnit (Fig. 1A and data not shown), a density that was low enough to avoid extensive cell-cell contact [8], [9].

**Fig. 2:**
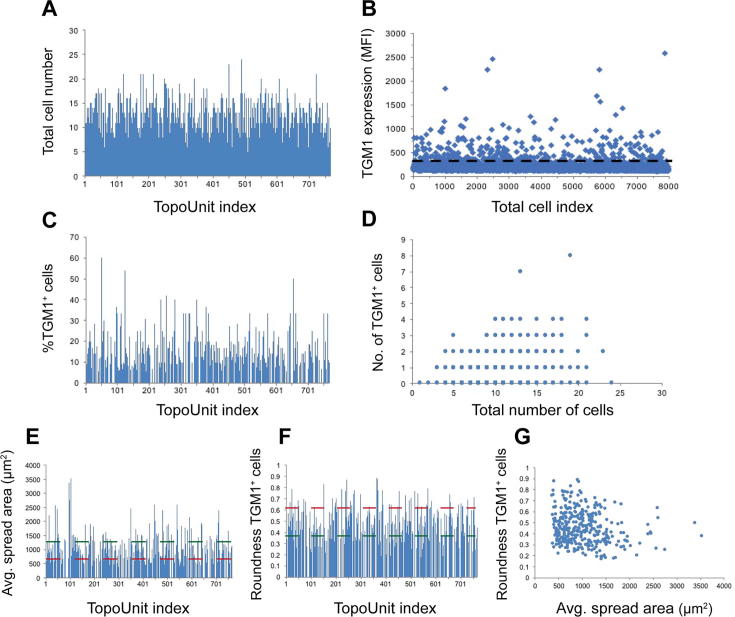
Quantification of differentiated cells. A) Quantification of total cell number on a quarter of a Topochip. Each TopoUnit is assigned a number, which corresponds to the TopoUnit index numbers in A, C and E–F. B) The expression of TGM1 in individual cells on the TopoChip shown in A. Cells above the dashed black line are TGM1^+^ (MFI > 270) and therefore differentiated. MFI > 270 represents a fluroescence intensity higher than that of a negative control sample (stained with secondary antibody only). C) The percentage of TGM1^+^ cells on individual TopoUnits. D) The number of TGM1^+^ cells compared to the total number of cells on individual TopoUnits. E, F) Quantification of E) the average spread area (µm^2^) and F) the roundness (circularity statistic, 4π*Area/Perimeter^2^) of differentiated cells. In E–F) the top threshold represents median + 2SDs, whereas the bottom threshold represents E) median − 0.5SD (red) or F) median − 1SD (green). TopoUnits with the most spread and least round differentiated cells (above the green threshold in E and below the green threshold in F) were compared to surfaces with the least spread and most round differentiated cells (below the red threshold in E and above the red threshold in F). In F) the roundness is calculated by the formula: 4π*Area/Perimeter^2^. Here, a value close to 1 represents an almost perfectly round cell (with a similar cell area and perimeter) and a value close to 0 a cell that is extremely elongated (i.e. it has a very large perimeter as compared to its cell area). G) Dotplot showing the distribution of the average spread area and roundness of differentiated cells. Most cells that are spread have a roundness under 0.5. Results are from one representative experiment out of five independent experiments. MFI: median fluorescence intensity. SD: standard deviation. TGM: transglutaminase-1.

To assess the differentiation of NHKs on the topographies, cells were cultured for an additional 24 h, fixed and labelled with antibodies to the differentiation marker transglutaminase-1 (TGM1) [17]. Cells were counterstained with phalloidin to detect polymerized actin (F-actin) and DAPI to stain nuclei. After stem cell adhesion, most of the cells were morphologically homogenous (Fig. 1A). After 24 h, on the other hand, cells exhibited a wide range of morphologies, from fully rounded to elongated or fully spread (Fig. 1B-C). One hour after plating, typically ≤5% of attached cells expressed TGM1 (Fig. 1A), which is consistent with previous reports [6], [18], [19]. The proportion of TGM1-positive cells was generally higher after 24 h. For the majority of topographies as well as the flat substrate control, the proportion of TGM1-positive cells was typically around 10–15%, but for some topographies the proportion was as high as 50–60% (Fig. 1B-C and 2C), indicating that certain topographies had a stimulating effect on differentiation. On some topographies the differentiated cells were round (Fig. 1B), whereas on others they were fully spread (Fig. 1C). Some topographies had no differentiated cells after 24 h, suggesting that such topographies had an inhibitory effect on differentiation (Fig. 2C and 4F).

### 3.2 Quantification of differentiation and morphological phenotype

To quantify the observed differences in morphology and differentiation, TopoChips were divided into quarters and imaged on an Operetta high-content imaging system (Perkin Elmer). In total 5 TopoChips were analysed with 8001100 TopoUnits per quarter. Typically, 70–80% of the TopoUnits could be quantitated during imaging analysis. The remainder could not be scored due to air bubbles, debris or damage to the TopoUnits. All identified TopoUnits contained cells (Fig. 2A). Cell numbers 24 h after plating ranged from 5-25 cells per TopoUnit (Fig. 2A). This was similar to directly after seeding (Fig. 1 and data not shown), indicating that there had not been substantial detachment or proliferation. To quantify the number of TGM1^+^ cells on each topography we measured the median fluorescence intensity (MFI) of TGM1 in individual cells. The morphology of differentiated cells was quantified by measuring their average spread area (total TGM1^+^ area divided by the number of differentiated cells) and roundness (circularity statistic: 4π*Area/Perimeter^2^). Total cell number was also quantified. Quantifications were performed using Harmony (PerkinElmer).

To analyse the different TopoUnits, we first identified them based on the intensity of the brightfield channel. Individual TopoUnits were saved as a region of interest, after which cellular features could be quantified. Cells were initially identified based on cytoplasmic F-actin staining. F-actin regions were saved as another region of interest, after which the number of nuclei and the MFI of TGM1 labelling were quantified. After comparison to a secondary antibody control (where the primary antibody staining had been omitted), this provided information about the expression of TGM1 on TopoUnits and thus about whether cells were differentiated or not. To score cells as TGM1^+^, a cut-off was set based on an MFI > 271 (Fig. 2B). This was based on the staining observed with secondary antibody labelling only.

As can be observed in Fig. 2B, most cells on TopoChips were negative for TGM1 and thus remained undifferentiated. Typically, 40–60% of the TopoUnits contained differentiated cells (Fig. 2C). The differentiated cell fraction ranged from 5–60%, with most TopoUnits (including the flat surface control), having 5–30% TGM1^+^ cells (Fig. 2C), corresponding to the proportion of terminally differentiated cells in confluent bulk cultures [20]. As can be observed in Fig. 2D, the number of TGM1^+^ cells did not correlate (r^2^: 0.05798) with the total number of cells, suggesting that the observed differentiation was primarily caused by interaction with the topographies and not by an increase in cell density or cell-cell adhesion.

To quantify morphology differences between differentiated cells we measured the average spread area and roundness of differentiated cells. Spread area was calculated by dividing the total TGM1^+^ cell area per TopoUnit by the number of TGM1^+^ cells on each TopoUnit (Fig. 2E). Cell roundness was calculated by quantifying the circularity statistic (4π*Area/Perimeter^2^, Fig. 2F). As expected from previous studies [6], [7] most differentiated cells on TopoChips had a relatively small spread area and had remained rounded and confined (Fig. 2E). Nevertheless, we also observed that on a small proportion of TopoUnits (10–15%), cells were highly spread (average spread area >1500 μm^2^, Fig. 2E). A similar percentage of cells had a low circularity statistic (<0.35, Fig. 2F), indicating a non-round morphology. Most of the highly spread TGM1^+^ cells had a relatively low roundness score (<0.5, Fig. 2G), suggesting that some keratinocytes differentiated as highly spread cells, in contrast to previous reports [6], [7].

### 3.3 *In silico* prediction of differentiation and spreading

To investigate whether specific topographical properties [21] promoted the differentiation of round versus spread cells, we ranked TopoUnits that promoted differentiation based on the spread area and roundness of TGM1^+^ cells. We compared the properties of topographies with highly spread differentiated cells (>median + 2SDs, observations above the green dashed line in Fig. 2E, >1272) versus those with non-spread differentiated cells (<median−0.5SD, observations below the red dashed line in Fig. 2E, <670). We also compared topographies with round (>median + 2SDs, observations above the red line in Fig. 2F, >0.62) versus non-round (<median − 1SD, observations below the green line in Fig. 2F, <0.38) differentiated cells. Perfectly rounded cells have a circularity statistic of 1, while highly elongated cells have a roundness close to 0. Comparisons were made by binary classification modelling as previously described [8], [16]. A description of the topographical features used for these analyses is provided in File S1.

**Fig. 3:**
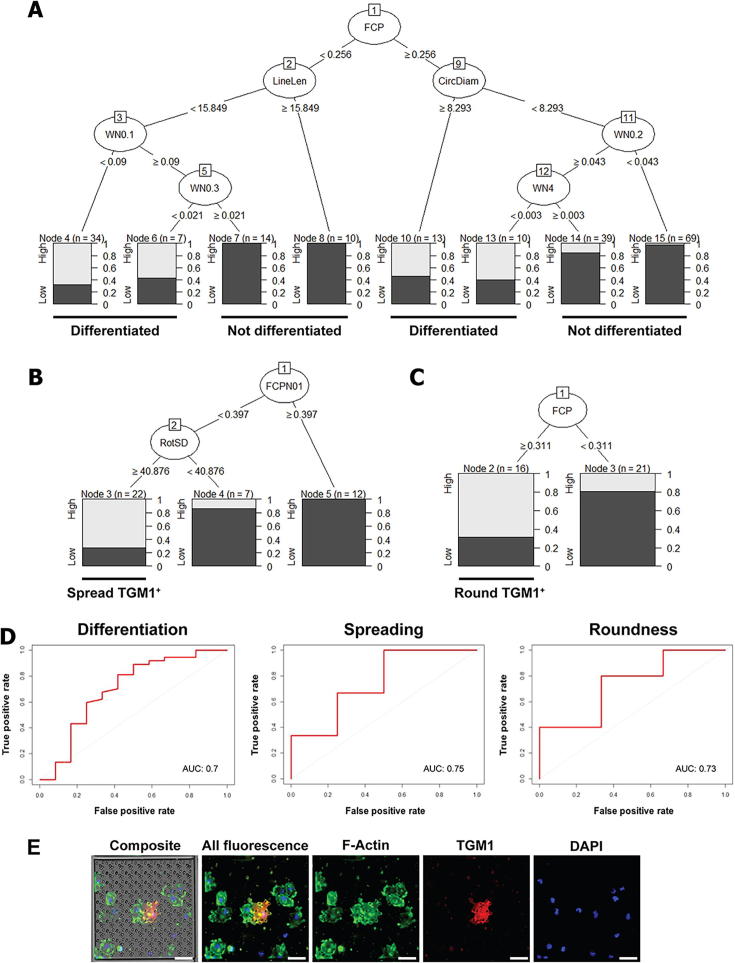
Computational models to predict the differentiation of keratinocytes. A-C) Classification trees showing the proportions of TopoUnits with A) differentiated cells, B) spread differentiated cells (above the top threshold in Fig. 2E) and C) differntiated cells that are round (cells above the top threshold in Fig. 2F), when separating for topographical parameters (described in File S1). D) ROC curves and AUCs for the models shown in A-C). Results are based on five independent experiments. Hits were ranked and selected per individual experiment. Light grey (and the annotation ‘high’) represents TopoUnits that were top hits from experiments. Dark grey (and the annotation ‘low’) represents bottom hits. FCP: proportion of pixels covered by topographies, LineLen: line length, CircDiam: circle diameter, WNx: the fraction of energy in the signal with wavenumber X, FCNP01: FCP with computationally added variation to account for more potential experimental variation, RotSD: standard deviation of the angle under which topography primitives are placed within the topography feature; a measure of topography irregularity (also see File S1). AUC: area under the curve. E) A TopoUnit surface that was a top hit in B (FCPN01: 0.0989901, RotSD: 162.428) and a bottom hit in C (FCP: 0.152628). Images show a differentiated cell that was highly spread. Scale bars in E represent 50 µm.

The results of our comparisons are shown in Fig. 3. The data for all TGM1^+^ cells, regardless of cell shape, is shown in Fig. 3A. Topography classification based on the spread area of differentiated cells is shown in Fig. 3B, while classification based on their roundness is shown in Fig. 3C. Top hits (labelled ‘high’ and in light grey) are topographies with the most differentiated cells (Fig. 3A), the most spread differentiated cells (Fig. 3B) or the most round differentiated cells (Fig. 3C). Bottom hits (labelled ‘low’ and in dark grey) are topographies with the fewest differentiated cells (Fig. 3A), the least spread (Fig. 3B) or the least round differentiated cells (Fig. 3C). The number (n) of TopoUnits in each category is shown for each node.

Several topographical features predicted differentiation irrespective of cell shape (Fig. 3A). The most important were: topography coverage (indicated by the FCP statistic), the length of line elements within topography features and the diameter of circle elements within topography features. When there was a high topography coverage (FCP ≥ 0.256 out of 1.000) circle diameter was important, with a large circle diameter (≥8.293 µm) resulting in more differentiation as compared to a smaller circle diameter (<8.293 µm). With a low topography coverage (FCP < 0.256), on the other hand, line length was more important. Here, short line length (<15.849 µm) was more supportive of differentiation than a longer line length (≥15.849 µm). Additionally, wavelength properties (which represent a combination of spatial properties converted into a single numerical value, File S1) could be used to further distinguish TopoUnits with more efficient differentiation, but only after initial separation based on line length, circle diameter and topography coverage. This indicates that specific topography properties, i.e. the number of topographies present on a TopoUnit and the size of certain elements within these topographies, affects the differentiation of epidermal stem cells. Some properties promote differentiation, while others are inhibitory.

With regards to the differentiation of spread cells, there were two predictive topographical parameters (Fig. 3B). One was again the coverage of topographies over the TopoUnit, this time represented by the FCPN01 statistic. This is highly similar to the FCP statistic, but calculated slightly differently. Both statistics separated the differentiation of spread versus non-spread cells, but FCPN01 was slightly better. The FCP statistic is computed based on the theoretical coverage that the topographies should have based on their design, while FCPN01 has more theoretical data points added to it and, hence, allows for more potential variation (e.g. due to minor damage/variations to the TopoChips) [File S1]. As shown in Fig. 3B, differentiated cells on topographies with a high coverage (≥0.397) were likely to be less spread than differentiated cells on topographies with a lower coverage (<0.397). The second parameter to separate spread from non-spread differentiated cells was RotSD (standard deviation of the rotation of topographical primitives, File S1). This is an indicator of irregularity within the topography feature (File S1). RotSD is calculated based on the angle under which the topography primitives (the circles, triangles and rectangles that make up a topography) are placed within the topography. Cells on topographies with a high degree of irregularity (RotSD ≥40.876°) were more likely to differentiate as spread cells compared to cells on topographies with a lower degree of irregularity (RotSD <40.876°, Fig. 3B).

For round differentiated cells, topography coverage (FCP) was also predictive (Fig. 3C). Here, TopoUnits with a high topography coverage (≥0.311) were more likely to contain differentiated cells that were round as compared to TopoUnits with a lower topography coverage (<0.311). This is the opposite of what we observed for spread cells (Fig. 3B), suggesting an inverse correlation between features that promote the differentiation of round and spread cells. Also, the FCP cut-off for round differentiated cells in Fig. 3C approaches the cut-off computed in Fig. 3A. This suggests that topographies in Fig. 3A that have an FCP of ≥0.256 (out of 1.0) and that contain large circular elements (circle diameter ≥8.293 µm) are likely to contain round differentiated cells. Similarly, topographies that have an FCP < 0.256 and that contain short line elements (line length <15.849 µm) are likely to contain spread differentiated cells. This suggests that small line length in combination with low topography coverage is important for the differentiation of spread cells, whereas circle diameter and high topography coverage is important the differentiation of round cells. This is consistent with published data on the differentiation of round cells [6], [7], as cells on a TopoUnit with higher topography coverage are more likely to experience restricted spreading compared to cells on a TopoUnit with low topography coverage.

Computing the receiver operating characteristic (ROC) curves for our predictive models (Fig. 3D) gave areas under the curves (AUCs) of: 0.7 (for the model shown in Fig. 3A), 0.75 (for Fig. 3B) and 0.73 (for Fig. 3C). Therefore, our models are reasonably accurate in predicting the observed phenotypes. The models for spread area and roundness could potentially be biased by a relatively small sample size, but the fact that they seem inversely correlated makes this unlikely. It is important to note that although we used binary classification tree algorithms to analyse the top and bottom hits from the TopoChip screen (Fig. 3), we did generate data for the full range of topographies and cell behaviours (Fig. 2).

Together, our results demonstrate that micron-scale topographies can direct epidermal stem cell morphology and differentiation. Furthermore, we show that NKHs can differentiate in a spread morphology, in contrast to previous observations [6], [7], [19]. An example of a TopoUnit that supports the differentiation of spread cells is shown in Fig. 3E.

### 3.4 Creation of topographies on a large scale

**Fig. 4:**
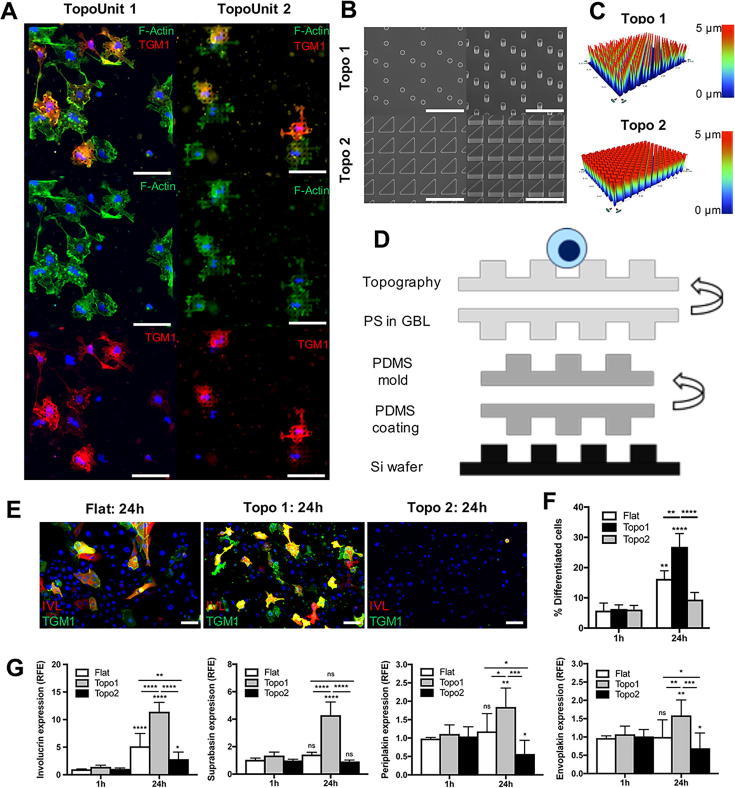
Validation of hits using fabricated polysterene topographies. A) Fluorescent microscopic images (20× objective) from TopoUnits that were chosen for further validation based on the analyses presented in Fig. 3. The first row represents composite images of all fluorescent channels; the second row an overlay of F-Actin (green) and DAPI staining (blue); and the last row an overlay of TGM1 (red) and DAPI staining. The first column represents the TopoUnit on which topography 1 was based. The second column represents the TopoUnit on which topography 2 was based. B) Scanning electron microscopy (SEM) images and C) height profiles of the fabricated topographies. In B) the first column represents top-view SEM images, while the second column represents images from a tilted (30°) view. D) Schematic overview of production of polystyrene (PS) topographies. Production starts with a silicon (Si) wafer, which is coated with polydimethylsiloxane (PDMS) to create a negative mould of the topographies after curing (>5h at 80 °C). The negative mould is then coated with PS that is dissolved in γ-butyrolactone (GBL). Upon evaporation of the solvent (4 h at 95 °C, followed by >12 h at 150 °C) this creates solid PS topographies that can be used for cell culture. E) Fluorescent microscopic images (20x) showing staining for TGM1 (green) and IVL (red) on flat and topography surfaces after 24 h of culture. Images represent overlays of DAPI (blue), TGM1 (green) and IVL (red). F) Quantification of the proportion of differentiated cells after 1 h versus 24 h of culture. G) Relative expression of the differentiation markers IVL, suprabasin, envoplakin and periplakin after 24 h of culture as determined by qRT-PCR. In F) differentiated cells are cells positive for TGM1. Scale bars represent 50 μm in A and E and 20 μm in B. Results in F and G are from 3 independent experiments. Results in A and E are representative images from five and three independent experiments, respectively. ns: not significant, *p < 0.05, **p < 0.01, ***p < 0.001, ****p < 0.0001 as determined by two-way ANOVA test (including Tukey correction for multiple comparisons). TGM1: transglutaminase-1, IVL: involucrin. RFE: relative fold expression. Topo1: topography 1, Topo2: topography 2. Si: silicon, PS: polystyrene, GBL: γ-butyrolactone, PDMS: polydimethylsiloxane.

To analyse the differentiation of spread cells in more depth, we developed a platform on which larger numbers of cells could be analysed. To do this, we selected one of the top hits from our initial TopoChip screen, which was also predicted from the computational analysis to support the differentiation of highly spread cells (TopoUnit 1; Fig. 4A). This topography (TopoUnit 1) had an FCP below 0.256, an FCPN01 below 0.397 and a RotSD above 40.876. Based on the analysis in Fig. 3A, B, this topography is predicted to promote differentiation of spread cells (i.e. FCP < 0.256, LineLen < 15.849, Wn0.1 < 0.09). We also included a topography that supported the differentiation of spread cells in one out of five experiments (66% TGM1^+^; Fig. 4A), but that was not predicted to do so based on the computational analysis (TopoUnit 2; bottom panel, Fig. 4B). It had an FCP < 0.256, LineLen < 15.849, Wn0.1 > 0.09, Wn0.3 > 0.021, FCPN < 0.397 and RotSD < 40.876. Therefore, we hypothesized that, if our data and models were correct, the replica of TopoUnit 1 should promote the differentiation of spread cells, whereas the replica of TopoUnit 2 should not.

Based on the dimensions of these topographies a silicon wafer template was fabricated by a nanofabrication company (Kelvin Nanotech). When replicas were made, one topography (topography 1) was found to faithfully replicate the original TopoChip structure (TopoUnit 1), whereas the replica of TopoUnit 2 was slightly different. Replication of the latter resulted in triangular, as opposed to the original bean-shaped, topographies (topography 2, Fig. 4B-C). The height profile of both topographies (∼5 µm) matched that of original TopoChip structures (Fig. 4C).

To produce topographies for larger scale experiments, the Si wafer was coated with polydimethylsiloxane (PDMS). After curing (>4h at 80 °C), this gave a negative mould of the topographies. This was then coated with polystyrene (PS) using benchtop soft-lithography [11]. For this, PS was first dissolved in the solvent γ-butyrolactone (GBL, 25 wt%). PDMS moulds were then coated with PS in GBL and the solvent was evaporated (4 h at 95 °C followed by >12 h at 150 °C) as described [11]. For an overview of the replica production see Fig. 4D. The coating method leaves only trace amounts of the solvent (<0.6%) behind on the topographies [11]. GBL was selected because it allows for complete dissolution of PS, while minimizing the swelling of the PDMS mould, thereby keeping the topography structure intact [11]. Since PS does not bind to PDMS, PDMS moulds could easily be peeled off after evaporation of the GBL. The PS topographies were air-plasma treated for 2 min and sterilised with 70% ethanol for 5 min prior to cell seeding.

We next computed the topographical properties of the fabricated surfaces using the same approach as for our TopoUnits. The properties of topography 1 were similar to the original TopoChip structure: FCP: 0.05875 (original: 0.09155), Wn0.1: 0.034267 (original: 0.037439), Wn0.2: 0.211541 (original: 0.173811), Wn0.3: 0.073330 (original: 0.027321), Wn4: 0.006481 (original: 0.014019). The properties of topography 2, on the other hand, were slightly different compared to the original: FCP: 0.3689 (original: 0.2083), Wn0.1: 0.289127 (original: 0.391952), Wn0.2: 0.063044 (original: 0.016717), Wn0.3: 0.048413 (original: 0.050569), Wn4: 0.002867 (original: 0.020291). This matches the altered appearance of topography 2 by SEM (Fig. 4B). The line length and circle diameter of topographies 1 and 2 could not be calculated, as these are based on the dimensions of the TopoUnit primitives chosen by *in silico* selection. Nonetheless, considering the structure of topography 1 is similar to the original TopoChip structure and has a low Wn0.1 (<0.09), we predicted that cells on this substrate would behave in a similar way to cells on the original structure (which has a line length of ∼11.46 and RotSD of 173.957) and would differentiate as spread cells. In the case of topography 2, we could not predict how the cells would respond, given the deviation from the original TopoUnit structure.

To evaluate the topographies experimentally, cells were seeded under the same conditions as on TopoChips, fixed and labelled with antibodies to TGM1 and IVL. This showed that Topography 1 stimulated the differentiation of spread cells (p < 0.0001, two-way ANOVA test), while topography 2 did not (Fig. 4E–F). The overlap between the two differentiation markers was ∼82% (Fig. 4E and data not shown), with IVL labelling slightly fewer cells (26.6% in Fig. 4E) compared to TGM1 (32.5% in Fig. 4E). When compared to a flat substrate, there were more differentiated cells on topography 1 after 24 h, whereas there were fewer on topography 2 (Fig. 4E–F).

The results of the immunofluorescence experiments were confirmed by quantitative (q)RT-PCR analysis (Fig. 4G). Cells on topography 1 and a flat control substrate showed an upregulation of all differentiation markers (IVL, suprabasin, periplakin and envoplakin) after 24 h, as compared to directly after seeding. Cells on topography 2, however, exhibited a reduction in periplakin and envoplakin, while slightly upregulating IVL, confirming their reduced differentiation potential. In addition, cells on topography 1 expressed higher levels of differentiation markers than cells on the other substrates. Cells on topography 2, on the other hand, had lower levels of the differentiation markers, both compared to cells on topography 1 and cells on the flat substrate.

**Fig. 5:**
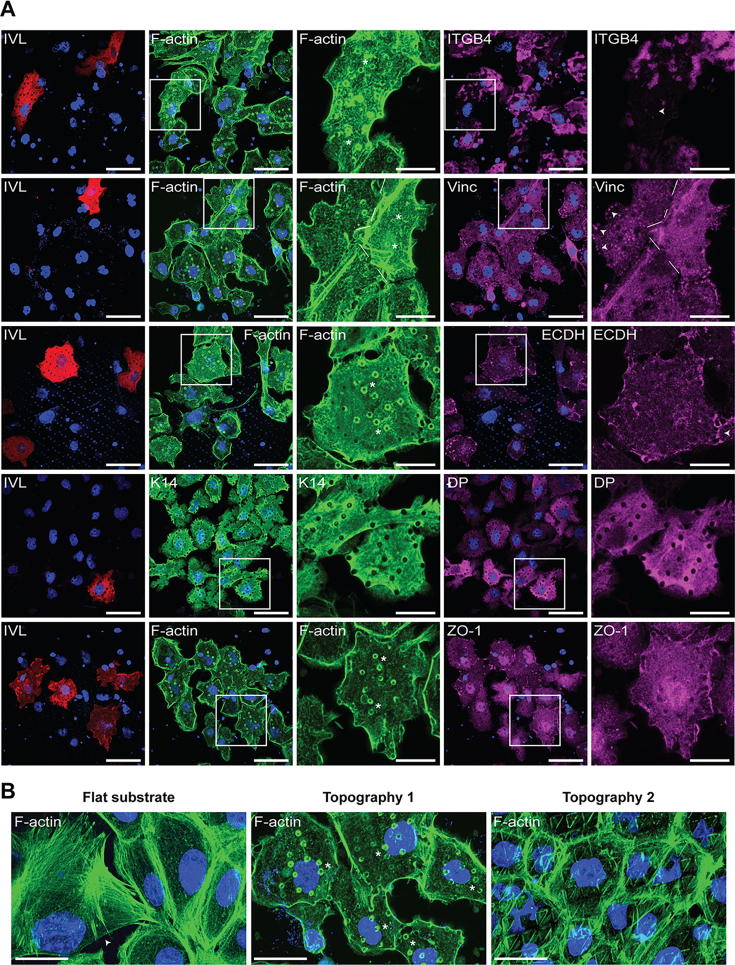
Actin rings, but not cell adhesion molecules, localize to the pillars of topography 1. A) Fluorescent microscopic images (63× objective) of involucrin (first column), F-actin or Keratin-14 (second and third columns) and indicated adhesion molecules (fourth and fifth columns). The first, second and fourth columns are counterstained with DAPI (blue). The third and fifth columns are zoom-in images of columns two and four, respectively, and represent single channel images of the indicated labels. Arrows indicate a lack of ITGB4 staining (row 1), the presence of focal adhesions (row 2) and adherens junctions (row 3), respectively. None of these markers localized to the pillars of topography 1. Asterisks show the localization of actin rings around the pillars. Scale bars represent 50 μm for columns 1, 2 and 4 and 20 μm for columns 3 and 5. B) F-actin staining (63× objective) of cells on flat substrate and topographies 1 and 2. The arrow indicates the presence of a contaminating J2-3T3 feeder cell (that contains actin stress fibers) on the flat surface. Asterisks indicate actin rings around the pillars of topography 1. Scale bars are 20 μm. Results are representative of three independent experiments. At least fifty cells were analysed per condition, including at least 10 differentiated cells. IVL: involucrin, K14: keratin-14, ITGB4: integrin-β4, Vinc: vinculin, ECDH: E-Cadherin, DP: desmoplakin, ZO-1: zonula occludens/tight junction protein-1. F-actin: polymerized actin.

The differentiation of spread cells on topography 1 validated our predictive model. In contrast, topography 2 seemed to have an inhibitory effect on differentiation. The cells that did differentiate on topography 2 had a mixture of round and spread morphologies. Cells on flat substrates could differentiate as spread cells, but typically tended to form stratifying colonies rather than differentiating as individual cells attached to the substrate (Fig. 4E, 5B and data not shown). Therefore, topography 1 presents a unique platform to specifically study the differentiation of spread cells.

### 3.5 Distribution of adhesion molecules

To see what could be driving the differentiation of spread cells, we stained NHKs on topography 1 with antibodies to various cell-cell and cell-substrate adhesion markers (Fig. 5A; Table S2). This showed that differentiated spread cells had lower levels of integrin-β4 (ITGB4), a marker of hemidesmosomes [22], than undifferentiated cells. There was no concentration of ITGB4 around the topographical features in either differentiated or undifferentiated cells. In addition, cells failed to cluster vinculin, a marker for focal adhesions [23] around the topographies (Fig. 5A). Similarly, no E-cadherin (adherens junctions) [24], desmoplakin (desmosomes) [25] or ZO-1 (tight junctions) [26] staining was localised to the topographies. Thus the differentiation of spread cells on topographies was not associated with accumulation of cell-cell adhesion or cell substrate molecules around the topographical features.

We did, however, observe an increased concentration of F-actin around the pillars of topography 1 (Fig. 5A, Fig. S2). This manifested itself as F-actin rings and was present in almost all the differentiated cells (97%; 37 out of 38 analysed cells). Cells negative for IVL showed a similar staining pattern, albeit less pronounced (91%; 187 out of 205 analysed cells). Keratin-14, on the other hand, did not show such staining, suggesting that there is a selective accumulation of F-actin rings around the pillars of topography 1. No actin rings were observed in cells on flat substrates or on topography 2. However, triangular patterns of actin assembly were sometimes observed on topography 2, indicating a potential response of the actin cytoskeleton to those topographical features as well (Fig. 5B).

### 3.6 Role of actin polymerization and actomyosin contractility

**Fig. 6:**
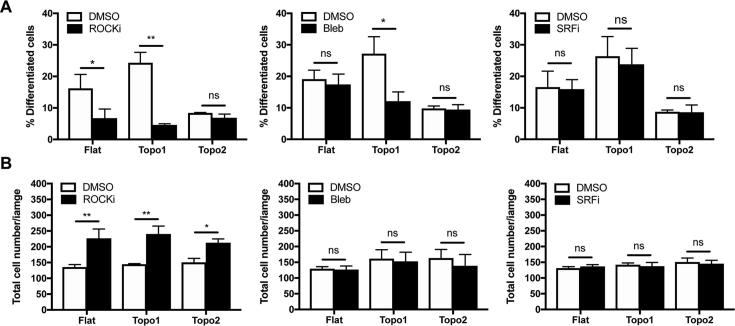
The effects of pharmacological inhibitors. A) Quantification of the percentage of differentiation (% TGM1^+^ cells) after 24 h of culture. Cells were cultured in FAD medium containing either 0.1% DMSO or ROCK inhibitor Y-27632 (left graph, 10 μM), blebbistatin (middle graph, 25 μM) or SRF inhibitor CCG-1423 (right graph, 1 μM). B) The total cell number (TGM1^+^ and TGM1^−^) per analysed image for cells cultured as in A. Results are from three independent experiments. Inhibitors were dissolved in DMSO at a concentration of 1000×, so that the concentration of DMSO in the final culture medium was 0.1%. ns: no significant difference, *p < 0.05, **p < 0.01 as determined by two-way ANOVA test (including Tukey correction for multiple comparisons). ROCKi: Rho-associated protein kinase inhibitor Y-27632, SRFi: serum response factor inhibitor CCG-1423, Bleb: blebbistatin, DMSO: Dimethyl sulfoxide.

Since F-actin is reported to be a regulator of epidermal differentiation [6], we hypothesized that the actin rings that formed on topography 1 might play a role in promoting the differentiation of spread cells, either via an increase in actin polymerization or actomyosin contractility. To explore this, we treated cells with pharmacological inhibitors and measured the proportion of TGM1^+^ cells 24 h after seeding (Fig. 6). We compared cells on topographies 1 and 2 and cells plated on a flat surface. Treatment with Rho-associated protein kinase (ROCK) inhibitor Y-27632 (ROCKi; 10 μM), which is known to inhibit both actin polymerization [6] and actomyosin contractility [27], inhibited keratinocyte differentiation on both topography 1 and the flat control (Fig. 6A). Differentiation on topography 2, on the other hand, was unaffected. ROCKi also increased the number of cells on each substrate (Fig. 6B), indicating that the cells had undergone more rounds of cell division. CCG-1423 (1 μM) treatment to inhibit serum response factor (SRF), a factor known to be required for keratinocyte differentiation on micro-patterned islands [6], [28], had no effect on cells on topography 1, topography 2 or flat substrates (Fig. 6A-B).

Treatment with the myosin II inhibitor blebbistatin (25 μM) also affected differentiation (Fig. 6A). However, unlike ROCKi, its inhibitory effect was specific to topography 1, as cells on both the flat substrate and topography 2 were unaffected. An increase in cell number was not observed after blebbistatin treament (Fig. 6B), indicating that blebbistatin inhibits differentiation without affecting proliferation. The specific effect on spread cells on topography 1 is consistent with previous studies showing that blebbistatin, unlike ROCKi, does not affect the differentiation of round cells [6].

Together, these results suggest that the actomyosin cytoskeleton mediates the differentiation of spread cells in response to micron-scale topographies and that the mechanisms by which round and spread cells differentiate are different.

## 4 Discussion

Cell-cell and cell-ECM interactions are key components of the epidermal stem cell niche [2], [4]. We have previously shown that restricted spreading on ECM-coated micropatterned islands, soft hydrogels and gold nanodots triggers the terminal differentiation of keratinocytes [7]. We have also shown that substrates that mimic the undulations of the human epidermal-dermal junction control stem cell patterning [29]. However, the response of keratinocytes to micron-scale topographical features has not been examined previously.

Here, we have used a high-throughput method to screen the effect of over 2000 different topographies on the differentiation of individual human epidermal stem cells. We show that specific topographies, those with a short line length or a large circle diameter (depending on the topography coverage and Wn statistic of the topographies) support the differentiation of keratinocytes after 24 h of culture. Furthermore, the topographical coverage (FCP) and degree of irregularity within the topographies (RotSD) affect whether a differentiated cell will be spread or rounded.

From the results of the screen we have designed a topography that promotes the differentiation of spread cells (topography 1) and a substrate that reduces differentiation (topography 2). These topographies were scaled up to cover the area of individual wells in a 6-well plate, allowing for a more detailed characterisation of differentiated cells that are spread. E-Cadherin and desmoplakin are expressed at higher levels in differentiated epidermal cells than in stem cells [30], but these markers did not accumulate around the pillar features of topography 1. Based on immunofluorescence staining and inhibitor experiments, we suggest that the responsiveness to topography 1 is mediated by the assembly of actomyosin rings and associated actomyosin contractility. Rho kinase activity was required for differentiation on both topography 1 and flat substrates and promoted proliferation. In contrast, inhibition of Myosin II specifically reduced differentiation on topography 1, without affecting proliferation. Differentiation on topography 1 is distinct from the differentiation response on micropatterned islands, which requires serum response factor [6]. We speculate that formation of actomyosin rings plays a role in the differentiation of spread cells. Mechanistically, recruitment of focal adhesions or hemidesmosomes, or clustering of cell-cell adhesion molecules is not required.

In summary, we have found that 1) substrate topography affects epidermal stem cell behaviour; 2) depending on the type of topography, differentiation can be either promoted or inhibited; 3) specific topographies promote differentiation of spread cells via a mechanism that is blocked by treating cells with ROCKi or blebbistatin. Further studies are necessary to elucidate the downstream signalling pathways. It will also be interesting to discover whether they are altered in skin conditions such as psoriasis in which the balance between proliferation and differentiation is disturbed.

## Figures and Tables

**Supplementary figure 1:**
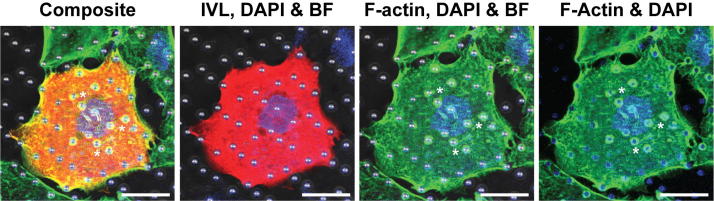


## References

[1] Hsu Y.C., Li L., Fuchs E. (2014). Emerging interactions between skin stem cells and their niches. Nat. Med..

[2] Watt F.M. (2016). Engineered microenvironments to direct epidermal stem cell behavior at single-cell resolution. Dev. Cell..

[3] Miroshnikova Y.A., Le H.Q., Schneider D., Thalheim T., Rübsam M., Bremicker N., Polleux J., Kamprad N., Tarantola M., Wang I., Balland M., Niessen C.M., Galle J., Wickström S.A. (2018). Adhesion forces and cortical tension couple cell proliferation and differentiation to drive epidermal stratification. Nat. Cell Biol..

[4] Lane S.W., Williams D.A., Watt F.M. (2014). Modulating the stem cell niche for tissue regeneration. Nat. Biotechnol..

[5] Hirsch T., Rothoeft T., Teig N., Bauer J.W., Pellegrini G., De Rosa L., Scaglione D., Reichelt J., Klausegger A., Kneisz D., Romano O., Seconetti A.S., Contin R., Enzo E., Jurman I., Carulli S., Jacobsen F., Luecke T., Lehnhardt M., Fischer M., Kueckelhaus M., Quaglino D., Morgante M., Bicciato S., Bondanza S., De Luca M. (2017). Regeneration of the entire human epidermis using transgenic stem cells. Nature.

[6] Connelly J.T., Gautrot J.E., Trappmann B., Tan D.W.M., Donati G., Huck W.T.S., Watt F.M. (2010). Actin and serum response factor transduce physical cues from the microenvironment to regulate epidermal stem cell fate decisions. Nat. Cell Biol..

[7] Trappmann B., Gautrot J.E., Connelly J.T., Strange D.G.T., Li Y., Oyen M.L., Cohen Stuart M.A., Boehm H., Li B., Vogel V., Spatz J.P., Watt F.M., Huck W.T.S. (2012). Extracellular-matrix tethering regulates stem-cell fate. Nat. Mater..

[8] Unadkat H.V., Hulsman M., Cornelissen K., Papenburg B.J., Truckenmuller R.K., Carpenter A.E., Wessling M., Post G.F., Uetz M., Reinders M.J.T., Stamatialis D., van Blitterswijk C.A., de Boer J. (2011). An algorithm-based topographical biomaterials library to instruct cell fate. Proc. Natl. Acad. Sci..

[9] Reimer A., Vasilevich A., Hulshof F., Viswanathan P., Van Blitterswijk C.A., De Boer J., Watt F.M. (2016). Scalable topographies to support proliferation and Oct4 expression by human induced pluripotent stem cells. Sci. Rep..

[10] Zhao Y., Truckenmuller R., Levers M., Hua W.S., de Boer J., Papenburg B. (2017). High-definition micropatterning method for hard, stiff and brittle polymers. Mater. Sci. Eng. C..

[11] Wang Y., Balowski J., Phillips C., Phillips R., Sims C.E., Allbritton N.L. (2011). Benchtop micromolding of polystyrene by soft lithography. Lab Chip.

[12] Jones P.H., Watt F.M. (1993). Separation of human epidermal stem cells from transit amplifying cells on the basis of differences in integrin function and expression. Cell.

[13] Rheinwald J.G., Green H. (1975). Serial cultivation of strains of human epidemal keratinocytes: the formation keratinizin colonies from single cells. Cell.

[14] Connelly J.T. (2012). Terminal differentiation of human epidermal stem cells on micro-patterned substrates. Methods Mol. Biol..

[15] Livak K.J., Schmittgen T.D. (2001). Analysis of relative gene expression data using real-time quantitative PCR and the 2-ΔΔCT method. Methods.

[16] Hulsman M., Hulshof F., Unadkat H., Papenburg B.J., Stamatialis D.F., Truckenmüller R., Van Blitterswijk C., De Boer J., Reinders M.J.T. (2015). Analysis of high-throughput screening reveals the effect of surface topographies on cellular morphology. Acta Biomater..

[17] Thacher S.M., Rice R.H. (1985). Keratinocyte-specific transglutaminase of cultured human epidermal cells: Relation to cross-linked envelope formation and terminal differentiation. Cell.

[18] Mishra A., Oulès B., Pisco A.O., Ly T., Liakath-Ali K., Walko G., Viswanathan P., Tihy M., Nijjher J., Dunn S.J., Lamond A.I., Watt F.M. (2017). A protein phosphatase network controls the temporal and spatial dynamics of differentiation commitment in human epidermis. Elife.

[19] Totaro A., Castellan M., Battilana G., Zanconato F., Azzolin L., Giulitti S., Cordenonsi M., Piccolo S. (2017). YAP/TAZ link cell mechanics to Notch signalling to control epidermal stem cell fate. Nat. Commun..

[20] Read J., Watt F.M. (1988). A model for in vitro studies of epidermal homeostasis: Proliferation and involucrin synthesis by cultured human keratinocytes during recovery after stripping off the suprabasal layers. J. Invest. Dermatol..

[21] Hulshof F.F.B., Papenburg B., Vasilevich A., Hulsman M., Zhao Y., Levers M., Fekete N., de Boer M., Yuan H., Singh S., Beijer N., Bray M.A., Logan D.J., Reinders M., Carpenter A.E., van Blitterswijk C., Stamatialis D., de Boer J. (2017). Mining for osteogenic surface topographies: In silico design to in vivo osseo-integration. Biomaterials.

[22] Schaapveld R.Q.J., Borradori L., Geerts D., Van Leusden M.R., Kuikman I., Nievers M.G., Niessen C.M., Steenbergen R.D.M., Snijders P.J.F., Sonnenberg A. (1998). Hemidesmosome formation is initiated by the β4 integrin subunit, requires complex formation of β4 and HD1/plectin, and involves a direct interaction between β4 and the bullous pemphigoid antigen 180. J. Cell Biol..

[23] Humphries J.D., Wang P., Streuli C., Geiger B., Humphries M.J., Ballestrem C. (2007). Vinculin controls focal adhesion formation by direct interactions with talin and actin. J. Cell Biol..

[24] Gumbiner B., Stevenson B., Grimaldi A. (1988). The role of the cell adhesion molecule uvomorulin in the formation and maintenance of the epithelial junctional complex. J. Cell Biol..

[25] Green K.J., Gaudry C.A. (2000). Are desmosomes more than tethers for intermediate filaments?. Nat. Rev. Mol. Cell Biol..

[26] Umeda K., Ikenouchi J., Katahira-Tayama S., Furuse K., Sasaki H., Nakayama M., Matsui T., Tsukita S., Furuse M., Tsukita S. (2006). ZO-1 and ZO-2 independently determine where claudins are polymerized in tight-junction strand formation. Cell.

[27] Kovács M., Tóth J., Hetényi C., Málnási-Csizmadia A., Seller J.R. (2004). Mechanism of blebbistatin inhibition of myosin II. J. Biol. Chem..

[28] Dubash A.D., Koetsier J.L., Amargo E.V., Najor N.A., Harmon R.M., Green K.J. (2013). The GEF Bcr activates RhoA/MAL signaling to promote keratinocyte differentiation via desmoglein-1. J. Cell Biol..

[29] Viswanathan P., Guvendiren M., Chua W., Telerman S.B., Liakath-Ali K., Burdick J.A., Watt F.M. (2016). Mimicking the topography of the epidermal-dermal interface with elastomer substrates. Integr. Biol..

[30] Simpson C.L., Patel D.M., Green K.J. (2011). Deconstructing the skin: cytoarchitectural determinants of epidermal morphogenesis. Nat. Rev. Mol. Cell Biol..

