# Supplementary material for: Micro-scaled topographies direct differentiation of human epidermal stem cells
Source: Acta Biomater. 2019 Jan 15;84:133–45. doi: 10.1016/j.actbio.2018.12.003 (PMC6336537; doi:10.1016/j.actbio.2018.12.003)
Supplement: Supplementary data 1 [file mmc1.docx]

**File S1: Explanation of topography properties.**

| **Feature properties** | **Description** |
| --- | --- |
| FCP | Fraction of the topography feature covered by primitive shapes. |
| FCPN0.1 | FCP with added noise N by formula 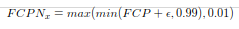 |
|  |  |
| CircDiam | The diameter of the circle primitive in topography features |
| LineLen | The length of the line element in topography features |
| WN0 **X** | The fraction of energy in the signal with wavenumber ca. **X** |

**FCP:**A measure of the topography coverage within a TopoUnit. Literally, the proportion of pixels within each well that is covered by a primitive shape (circle, triangle, rectangle). Since primitive shapes makeup the topography features, this statistic indicates the proportion of the well covered by topographies. For example: an FCP of 0.4 would mean that 40% of the TopoUnit is covered with topographies. The FCP ratio differs per topography.

**FCPN01:**This statistic is correlated to FCP. It is calculated in the same way, but when the FCPN01 is calculated more potential data points are considered in the analysis; not just the ones corresponding to the analysed topography features. This is to compensate for small errors in the fabrication procedure or due to minor damage to the TopoUnits. Therefore, the FCPN01 considers more variability in the data range. Ideally the FCP and FCPN01 would be the same, but this is not always the case (due to the above-mentioned reasons). In case of small changes in the TopoChip structure, the coverage of the topographies could slightly change and therefore the FCPN01 could be more specific than the FCP (since it accounts for more variation).

**RotSD:**A measure of the irregularity within a topography feature. It is calculated by taking the standard deviation of the angles under which each primitive is placed in the topography feature. This is calculated relative to the x-axis of a projected box around the feature (see image below). This box is not visible on the TopoUnit but is used as a grid to place features in the right position on the TopoChip (after *in silico* analysis). If there are many different angles under which primitive elements are placed in the topography feature, this will lead to a higher RotSD and a more irregular outline of the topography feature. Likewise, features with a lower RotSD will typically have a smoother/more uniform shape.

**LineLength:**The length (in micrometer) of line elements used in a topography feature.

**CircDiam:**The diameter (in micrometer) of circle elements used in a topography feature.

**Wn**
The Wn statistic is a mathematical statistic based on the discrete Fourier transformation (DFT). This gives a mathematical value to a topography feature based on various spatial properties (such as size/shape of the primitives, dimensions of the feature and the distribution/clustering of primitives within the topography feature, etc.). Features that have a high value for Wn0.1 often have the dimensions 10μm x 10μm and contain primitives clustered in the middle of the topography feature (often line and circle primitives). Features that have high values for Wn4 often contain small triangles that are well spread over the feature. Increasing Wn (Wn0.1 to Wn4) amongst other things goes together with a more equal spreading of primitives over the topography feature.
